# Anaemia and associated factors among children aged 6–59 months during the post-ebola period in Sierra Leone: a national cross-sectional survey- 2019

**DOI:** 10.1186/s13690-024-01290-9

**Published:** 2024-09-14

**Authors:** Linet M. Mutisya, Quraish Sserwanja, Kassim Kamara, Micheal Mazzi, Emmanuel Olal

**Affiliations:** 1 Programs Department, Relief International, Khartoum, Sudan; 2 Maternal and Child Health Project, Swedish Organization for Global Health, Mayuge, Uganda; 3 National Disease Surveillance Programme, Ministry of Health and Sanitation, Free town, Sierra Leone; 4 Programmes Department, Partners in Health, Freetown, Sierra Leone; 5 Yotkom Medical Centre, Kitgum, Uganda

**Keywords:** Anaemia, Children, Malnutrition, Sierra Leone

## Abstract

**Background:**

Anaemia is a global public health problem associated with early childhood adverse effects on mental, physical, and social development. Sierra Leone had made progress in reducing the prevalence of anaemia pre-Ebola period however this was affected by the Ebola epidemic which further strained an already struggling health system. Therefore, this study aimed to assess the prevalence and factors associated with anaemia during post-Ebola period among children aged 6–59 months in Sierra Leone.

**Methods:**

We analyzed data from the 2019 Sierra Leone demographic and health survey (SLDHS), a nationally representative cross-sectional study. We used data collected using a stratified two-stage cluster sampling design that resulted in the random selection of a representative sample of 13,872 households. A total sample of 3,459 children aged 6–59 months were included in the study. Multivariable logistic regression was used to calculate the adjusted odds ratios and corresponding 95% confidence intervals.

**Results:**

The prevalence of anaemia was 68.9%, that of mild anaemia was 35.8%, moderate anaemia was 30.3% and for severe anaemia was 2.8%. Children aged 6–36 months were 1.83 times more likely to have anaemia compared to those above 36 months, while boys 1.33 times more likely to be anaemic compared to girls. Children born in poor households, to mothers who had anaemia and had a history of fever had 65%, 85% and 38% increase in likelihood of childhood anaemia respectively. In addition, children living in rural areas and stunted were 1.55 and 1.38 times more likely to be anaemic respectively compared to those living in urban areas and not stunted. Children born to younger mothers (15–24 years) were 1.45 times more likely to be anaemic compared to older mother (35–49 years.

**Conclusion:**

The current study demonstrated the predominant existence of anaemia among children aged 6–59 months in Sierra Leone. Owing to the adverse effects of anaemia on the development of children in the future, there is an urgent need for effective and efficient remedial public health interventions to prevent further complications.

**Table:** 

**Text box 1. Contributions to the literature**
1. Literature has shown anaemia to have devastating effects on the cognitive and physical development of children and more so in resource-poor settings due to the prevalence of infectious diseases such as Malaria
2. Even though we found a slight improvement in the prevalence of anaemia during the post-Ebola period compared to the pre-Ebola period, it remains a major public health problem. This comparison brings out the inadequacies of the healthcare system
3. These findings provide key priority areas that implementation should be focused on to reduce the prevalence of Anaemia among children aged 6–59 months in Sierra Leone

## Background

Anaemia, characterized by low haemoglobin concentration, is a significant global public health problem [1–3]. It affects about 25% of the world’s population and approximately 43% of children below five years of age [4]. However, few countries are on track to meet the World Health Organization (WHO) target of reducing anaemia by 50% by 2025 [5]. Sub-Saharan Africa accounts for over 67% of anaemia cases globally, contributing to morbidity and mortality in women and children [4, 6].

Children are particularly vulnerable to anaemia due to physical and psychological development [7–10]. Anaemia leads to long-term cognitive and developmental delays, impacting school performance and future productivity [11]. Children with anaemia also have a compromised immune function, making them more susceptible to infectious diseases [11].

The causes of childhood anaemia are multifactorial, including micronutrient deficiencies, infections, parasitic infestation, and genetic disorders [12]. Inadequate iron intake, poor diet quality and quantity, hygiene practices, absorption issues, and chronic blood loss contribute to iron demands not being met [4, 7, 13]. Socioeconomic factors such as education, wealth, income, and sanitation are associated with anaemia prevalence. Effective interventions require interdisciplinary approaches and multi-sectoral collaboration [6, 14].

Sierra Leone’s post-Ebola period is crucial, as the country was recovering from a civil war that severely impacted the healthcare system [15]. Childhood anaemia in Sierra Leone is among the highest globally, with a prevalence of 76%, primarily caused by malaria, diarrhea, and respiratory infections [16]. Malaria prevalence among children under five is approximately 53% [17]. Anaemia is also prevalent among lactating women, affecting their ability to provide proper nutrition [18].

To address anaemia and other health issues post-Ebola, the Sierra Leone Government established the national anaemia working group, focusing on anaemia priorities [19]. There is a drive to reduce anaemia through malaria prevention, dietary diversification, micronutrient supplementation, and health education [19]. This study aims to investigate the prevalence and factors associated with anaemia in children aged 6–59 months during the post-Ebola period in Sierra Leone using the most recent nationally representative survey, SLDHS 2019.

## Methods

### Study design and participants

#### Data source

The SLDHS 2019 was a nationally representative cross-sectional study implemented by Statistics Sierra Leone (Stats SL) with technical assistance from ICF through the DHS Program funded by the United States Agency for International Development (USAID). Data collection took place between May and August 2019 [20].

### Study population and eligibility criteria

Administratively, Sierra Leone is divided into provinces which are further divided into districts, and finally enumeration areas (EAs) from the 2015 EA census frame [20]. A two-stage stratified cluster sampling design was used to select a random representative sample of households [20]. Stratification was achieved by separating districts into urban and rural areas and a total of 31 sampling strata were created, followed by selection of 578 EAs [20].

### Sample size

A fixed number of 24 households were further selected in every cluster through equal probability systematic sampling resulting in 13,872 households [20] The survey used a sampling frame from the 2015 population census. In the 2019 SLDHS, the primary sampling unit (PSU) was referred to as a cluster.

### Data collecting procedures

Anthropometric measurements were taken for adults and children in selected household by trained research assistants using standard instruments and results recorded in DHS questionnaires.

### Laboratory sample collection and hemoglobin analysis

#### Informed consent

(guardians gave consent for children) was sought for drawing blood samples to measure haemoglobin levels in selected households. Blood samples were then obtained via a finger prick (or a heel prick for children aged 6–11 months) and haemoglobin levels estimated in real-time with a portable HemoCue analyzer [20]. Results of anthropometry measurements and other biomarkers for men, women, and children were recorded using the biomarker questionnaire which was administered to only 50% of households selected for the male survey [20]. Parents or caretakers of children with a haemoglobin level below 8 g/dl were advised to take the child to a health facility for treatment and follow-up [20]. Out of the 9,899 children in the 2019 SLDHS, 5,937 did not have their haemoglobin levels assessed. Our analysis excluded children whose haemoglobin level results were missing (not present for testing or consent was not granted) or recorded as “Flagged cases” and therefore our final sample included 3,459 children aged 6–59 months. Written permission to access the whole SLDHS database was obtained through the DHS program website (https://dhsprogram.com/).

### Variables

#### Outcome variables

Anaemia was assessed using haemoglobin (Hb) level measured during the DHS survey. Here anaemia was defined as Hb < 11.0 g/dl. For this study, we further categorized anaemia as follows: mild (Hb 10.0 to 10.9 g/dl), moderate (Hb 7.0 to 9.9 g/dl) and severe (Hb < 7.0 g/dl) [20]. Haemoglobin levels were adjusted for the altitude in areas that were 1,000 m above sea level.

#### Independent variables

Independent variables were broadly grouped into child, parent and household-specific characteristics based on previous studies and availability in the SLDHS database. Maternal characteristics assessed included anaemia status (anaemic and not anaemic), level of education (no education, primary, secondary and tertiary), pregnancy status (pregnant and not pregnant), age in years (15–24, 25–34, 35–49), marital status (married and not married), working status (working and not working) and parity (less than 5 and 5 and above).

Household characteristics included wealth index (richest, richer, middle poorer and poorest), area of residence (urban and rural), number of household members (< 6 or 6 and above), sex of household head (female and male) and region (Northwest, Eastern, Western, Southern and Northern).

Child-specific characteristics included age in months (6–36 months and 37–59 months), sex (male and female), recent history of fever, upper respiratory tract infection (URTI), diarrhea and deworming in the last six months (yes and no). Nutritional status was assessed using z-scores based on the 2006 WHO Child Growth Standards for height-for-age (HAZ), weight-for height (WHZ) and weight-for-age (WAZ) [21, 22]. Stunting was defined as HAZ < -2 SD, wasting as WHZ < -2 SD and underweight as WAZ < -2 SD [21–23].

### Statistical analysis

We used the SPSS analytic software version 25.0 Complex Samples package for this analysis (IBM Corp. Released 2017. IBM SPSS Statistics for Windows, Version 25.0. Armonk, NY: IBM Corp.). To account for the unequal probability sampling in different strata, we weighted the data prior to analysis. Frequency distributions were used to describe the background characteristics of the children. Continuous variables for descriptive analysis were expressed in mean and standards deviation. Bivariable logistic regression was also conducted, and we present crude odds ratio (COR), 95% confidence interval (CI) and *p*-values. Independent variables found significant at a *p*-value less than 0.25 in the bivariable analysis were included in the multivariable model. Adjusted odds ratios (AOR), 95% Confidence Intervals (CI) and *p*-values were calculated with statistical significance level set at *p*-value < 0.05. Model fitness was assessed with the *Hosmer-Lemeshow test*. All variables in the model were assessed for collinearity, which was considered present if the variables had a variance inflation factor (VIF) greater than 5. However, none of the factors had a VIF above 3.

## Results

The mean age of children was 2.1 years with SD ± 1.3 years while mean Hb was 10.2 g per deciliter (g/dl) with SD ± 1.5 g/dl. The proportion of boys and girls in the study was equal (boys, 50.3%). Most children resided in rural areas (66.5%) and belonged to households headed by males (76.5%). Recent history of fever was observed in 18.4% of all children. About 2,382 children (68.9%) had anaemia with 30.3% (1,047), 35.8% (1,239) and 2.8% (95) having mild, moderate and severe anaemia respectively. Characteristics of study participants are shown in Table 1.

**Table 1:** Background characteristics of children aged 6–59 months during the post-Ebola period in Sierra Leone tested for aneamia

Characteristics	*N* = 3459	Percentages
**Mother’s Parity**		
Less than 5	2452	70.9
5 and above	1007	29.1
**Rural Residence**	2301	66.5
**Region**		
Western	581	16.8
Northwest	510	14.7
Eastern	815	23.6
Southern	829	24.0
North	725	21.0
**Household size**		
Less than 6	1347	39.0
6 and above	2111	61.0
**Working mothers**	2783	80.5
**Married mothers**	2976	86.0
**Mother’s education Level**		
Tertiary	95	2.7
Secondary Education	913	26.4
Primary Education	576	16.6
No Education	1875	54.2
**Wealth Index**		
Richest	502	14.5
Richer	607	17.6
Middle	705	20.4
Poorer	764	22.1
Poorest	880	25.5
**Mother’s age**		
15–24	924	26.7
25–34	1633	47.2
35–49	902	26.1
**Age of child**		
6–36 months	2042	59.0
37–59 months	1417	41.0
**Anaemic mothers (53 missing data)**	1661	48.0
**Mothers pregnant**	245	7.1
**Male Head Household**	2646	76.5
**Male children**	1741	50.3
**With recent fever history** **N**	637 148	18.4
**Recent diarrhea**	257	7.4
**Recent URTI**	500	14.4
**Recent deworming (6 months)**	2209	63.9
**Underweight**	687	20.2
**Stunting**	853	25.1
**Wasting**	165	4.8
**Children with Anaemia**	2382	68.9

### Factors associated with childhood anaemia

Results from multivariable logistic regression (Table 2) showed that wealth index, mother’s anaemia status and age, sex of the child, recent history of fever, residence and child’s age and stunting status were associated with childhood anaemia. Younger children (aOR = 1.83; 95% CI 1.55–2.15, *P* < 0.001) were 55% more likely to be anaemic compared to the older ones, while boys (aOR = 1.33; 95% CI 1.14–1.55, *P* < 0.001) were 33% more likely to be anaemic compared to girls. Household poverty (aOR = 1.65; 95% CI 1.02–2.67, *P* < 0.05), maternal anaemia (aOR = 1.85; 95% CI 1.53–2.25, *P* < 0.001), and a history of fever (aOR = 1.38; 95%CI 1.06–1.80, *p* < 0.005) were associated with 65%, 85% and 38% more likelihood of childhood anaemia respectively. Furthermore, chidren living in rural areas (aOR = 1.55; 95% CI 1.07–2.24, *P* < 0.05) were stunted (aOR = 1.38; 95% CI 1.09–1.76, *P* < 0.01), and born to younger mothers (15–24 years) (aOR = 1.45; 95% CI 1.09–1.93, *P* < 0.05) were 55%, 38% and 45% more likely to be anemic respectively.

**Table 2:** Predictors of anaemia among children aged 6–59 months during the post-Ebola period in Sierra Leone

Characteristics		Adjusted Model aOR(95%CI)	*P*-value
**Child’s age**	37–59 months	Ref	< 0.001
37–59 months	6–36 months	**1.83 (1.55–2.15)**	
**Child’s sex**	Female	Ref	< 0.001
	Male	**1.33 (1.14–1.55)**	
**Wealth index**	Richest	Ref	< 0.001
Richest	Richer	**1.76 (1.15–2.70)**	
Richer	Middle	1.51 (0.93–2.46)	
Middle	Poorer	**1.65 (1.02–2.67)**	
Poorer	Poorest	1.64 (0.96–2.82)	
Mother with anaemia*	Yes	**1.85 (1.53–2.25)**	< 0.001
Recent fever* **‘history**	Yes	**1.38 (1.06–1.80)**	0.005
**Residence**	Urban	Ref	< 0.001
	Rural	**1.55 (1.07–2.24)**	
**Mother’s age**	35–49	Ref	0.088
	25–34	1.10 (0.88–1.38)	
	15–24	**1.45 (1.09–1.93)**	
**Mother’s marital status**	Married	Ref	0.020
	Not Married	0.91 (0.71–1.16)	
**Mother’s education Level**	Tertiary	Ref	< 0.001
	Secondary Education	0.78 (0.45–1.35)	
	Primary Education	0.85 (0.48–1.50)	
	No Education	1.18 (0.68–2.04)	
**Region**	Western	Ref	< 0.001
	Southern	0.88 (0.56–1.38)	
	Northwest	1.21 (0.77–1.90)	
	North	1.07 (0.68–1.69)	
	Eastern	0.91 (0.59–1.41)	
**Mother’s parity**	Less than 5		0.635
	5 and above		
**Household size**	Less than 6		0.266
	6 and above		
Mother pregnant*	Yes		0.448
**Head Household**			0.007
Female	522 (21.9)	Ref	
Male	1860 (78.1)	1.18 (0.95–1.46)	
**Mother working** **N**			0.183
Yes Primary	1934 (81.2)	Ref	
No	448 (18.8)	1.10 (0.85–1.42)	
Recent diarrhea*	Yes	0.92 (0.66–1.29)	0.147
Recent URTI*	Yes	1.09 (0.81–1.45)	0.037
Underweight*	Yes	0.88 (0.66–1.17)	0.155
Stunting*	Yes	**1.38 (1.09–1.76)**	0.001

*The “No” category, not presented, is the reference

## Discussion

Childhood anaemia is a major public health concern in Sierra Leone. This study assessed the prevalence of, and factors associated with anaemia among children aged 6 to 59 months. The post Ebola prevalence (68.9%) of anaemia among Sierra Leonean children in this study was 11.1 and 7.4% points less compared to the pre-Ebola 2013 SLDHS prevalence (80%) and the 2013 SLMS prevalence (76.3%) respectively which shows a slight decline in the prevalence over the decade [13, 24]. According to the World Health Organization (WHO) prevalence cut off values for public health significance, this study’s finding would be classified as a severe public health problem despite the slight improvement realized post Ebola. This shows poor progress towards achieving the 2025 global nutrition targets and 2030 sustainable development goals [25].

Child’s age, sex and history of fever were significantly associated with childhood anaemia in the pre Ebola era [13], an association that also persisted in the post-Ebola era as shown in this study. Wealth index and residence were not significantly associated with childhood anaemia in the pre-Ebola analysis but showed a significant association in this post-Ebola era analysis. In addition, child stunting status and maternal factors such as age and anemia status were significantly associated with childhood anaemia in this study, though not analyzed in the pre-Ebola era. The country was still recovering from the devastating effects of the civil war that incapacitated its health system.

The high prevalence of anaemia in Sierra Leone could be explained by the fact that the country has one of the highest rates of malaria (a main cause of anaemia) transmission rates in Africa as reported in the 2013 SLMS, which worsened in the post-Ebola period [26]. Similar cross-sectional studies conducted in Ethiopia [6], Uganda [27] and Ghana [28] showed slightly lower rates compared to Sierra Leone.

This study found that younger children (6–36 months) were more likely to have anaemia compared to their older counterparts, consistent with other studies [29–31] that showed a significant decrease in the likelihood of anaemia with increase in age. The higher odds of anaemia in younger children could be driven by early cessation of breastfeeding and the concomitant introduction of inappropriate complementary foods (before or at six months of age), especially diets with inadequate bio-available iron [12, 13]. In addition, other factors such as low maternal iron reserve during pregnancy could lead to anaemia in their infants [32]. Generally, children are born with body iron reserves that aid in growth and protection from iron deficiency up to six months. Thereafter, the iron stores are gradually depleted necessitating introduction of food to complement breastfeeding which alone may not provide sufficient iron required during this period of rapid growth [33, 34].

Children with a recent history of fever were more likely to have anaemia compared to those with no recent history of fever. A similar study conducted in Bangladesh [4], identified fever as a common symptom of acute and chronic diseases which has been associated with lower haemoglobin levels and anaemia. Recent history of fever may indicate recent malaria, a disease characterized by the destruction of red blood cells and subsequent anaemia [33].

Boys were more likely to have anaemia compared to girls, findings also observed in similar studies from Kenya [35], Ghana [36] and Cameroon [37]. This could be related to the higher growth rate in boys and the complex interactions between type of diet, iron availability while breastfeeding and complementary feeding, growth velocity and iron loss through infection and infestation [38, 39].

Children from poor households (low SES) were more likely to have anaemia compared to those from better off households which was also observed in a study conducted in rural Ethiopia [40]. Affordability of diverse nutritious foods is often compromised in poor households which reflects food insecurity [33, 41, 42].

Children born to mothers with anaemia had 1.85 increased odds of anaemia compared to those born to mothers without, similar to studies conducted in Ethiopia and Bangladesh [4, 43]. When a child is born, breast milk forms the main source of nutrients at least for the first six months of life. However, body iron stores are reduced in children whose mothers had anaemia during pregnancy, predisposing them to childhood anaemia [14, 33]. Household poverty and associated inadequate dietary intake affects maternal nutrition requirements leading to anaemia, a contributing factor to both premature (children get majority of their iron stores from their mother during the last trimester of pregnancy) and low birthweight babies hence increasing the risk of childhood anaemia [33]. Mother’s level of education even though generally confounded with socio-economic status plays a major role in knowledge acquisition [44, 45].

Children born to younger mothers were 1.45 times more likely to have anaemia compared to those born to older mothers. Younger women from poor socio-economic households tend to get pregnant sooner after giving birth, mostly within six months, a period in which maternal iron stores are still recovering [4]. In addition, young mothers may not have the required experience and knowledge in proper childcare practices which may lead to child malnutrition [38]. These studies [4, 14, 46] showed a similar trend.

Children residing in rural areas were 1.55 times more likely to have anaemia compared to those residing in urban areas. Rural areas are characterized by unfavorable socio-economic status, lack of insufficient and safe drinking water and poor sanitation, a known risk factor for the intestinal parasite hookworm, diarrhea as well as environmental enteropathy (EE), a subclinical disorder of the small intestines. This may impair the absorptive function of the small intestine mucosa lining which may lead to anaemia. A meta-analysis of demographic health surveys conducted in low- and middle-income countries showed a positive association between children living in rural areas and anaemia compared to those in urban-poor areas [46].

Stunted children were more likely to suffer from anaemia possibly due to household food insecurity leading to inadequate dietary intake hence increasing the child’s susceptibility to both anaemia and stunting as depicted in the UNICEF conceptual framework for malnutrition [4, 34]. Furthermore, inadequate health facilities in rural areas pose a challenge in the provision of quality health care services such as nutrition counselling and immunization [38].

### Strengths and limitation

The main strengths of this study were the use of large, nationally representative samples and objectively measured Hb levels. The inclusion of various explanatory factors (child, maternal and household characteristics) might have improved the comprehensiveness of the study and enabled adjustment for potential confounders. However, this study has several limitations. First, owing to the cross-sectional study design, it was not possible to infer causality. Second, some of the variables’ data were based on respondents’ memory of past events which might have introduced recall bias. Third, the study did not account for the effects of dose, duration, and adherence of deworming, iron, and vitamin A supplementation, which might have also biased our findings. Fourth, data for the assessment of intestinal and blood parasite, Vitamin B12, folate which significantly affect the hemoglobin level of children and a major contributing factor of anemia in under-five children was not available for this study. Despite these limitations, we believe the findings of this study could serve as evidence basis for further studies on the determinants of childhood anaemia in Sierra Leone.

## Conclusion

This study shows a slight decrease in the prevalence of anaemia among children under five years in Sierra Leon, however, remains a major public health concern. Child related factors were mainly associated with anaemia during the pre-Ebola period, however more factors including household and maternal factors added to the list during the post-Ebola period as shown in this study. Nutrition stakeholders need to prioritize nutrition education programs and policies targeting rural younger mothers from poor households. Screening for anaemia in children with fever especially those aged 6–36 months and those stunted should also be considered. Therefore, there is need to design and scale up comprehensive health promotion and nutrition education interventions, with due emphasis on the multifactorial nature of anaemia, which may potentially reduce the burden of childhood anaemia in Sierra Leone.

## Data Availability

The data that support the findings of this study are available from MEASURE DHS website (URL: https://www.dhsprogram.com/data/available-datasets.cfm) but restrictions apply to the availability of these data, which were used under license for the current study, and so are not publicly available. Data are however available from the MEASURE DHS website (URL: https://www.dhsprogram.com/data/available-datasets.cfm) upon reasonable request and with permission of MEASURE DHS.

## Abbreviations

EA: Enumeration area
AOR: Adjusted Odds Ratio
CI: Confidence Interval
COR: Crude Odds Ratio
DHS: Demographic Health Survey
SDHS: Sierra Leone Demographic Health Survey
OR: Odds Ratio
WHO: World Health Organization
SPSS: Statistical Package for Social Science
